# Editorial: The UN international day of families: neurodegeneration as a result of genetic inheritance

**DOI:** 10.3389/fnagi.2023.1291613

**Published:** 2023-10-06

**Authors:** Iliyana Hristova Pacheva, Durga Attili, Stylianos Ravanidis

**Affiliations:** ^1^Department of Pediatrics and Medical Genetics, Plovdiv Medical University, Plovdiv, Bulgaria; ^2^Department of Pathology, University of Michigan Medical School, Ann Arbor, MI, United States; ^3^ECONCARE LP, Athens, Greece

**Keywords:** neurodegeneration, neurodegenerative diseases, genetic inheritance, Alzheimer's disease, Parkinson's disease, dementia, single nucleotide (nt) polymorphism (SNP), ischemic stroke

Neurodegenerative diseases (NDs) represent a group of heterogeneous conditions affecting the motor and cognitive functions of patients. Alzheimer's (AD) and Parkinson's disease (PD) for example may be categorized as distinct NDs, however common overlapping traits occur, such as neuronal loss and accumulation of aggregated or misfolded proteins (Ruffini et al., 2020). Moreover, common malfunctions in cellular homeostasis processes take place resulting in impaired protein dynamics (Jellinger, 2010). Therefore, identifying and delineating the contribution of genetic inheritance in the malfunction of these processes could improve the prognosis and the therapeutic management of these patients. In this Research Topic we aimed to collect articles that discuss new research findings over the genetic inheritance in NDs.

Gao et al. explored the importance of presenilin-1 (PSEN1) in the regulation of amyloid β-protein 1-42 (Aβ42) accumulation related to familial AD. The authors examined the efficacy of angiotensin-converting enzyme (ACE), responsible for the conversion of neurotoxic Aβ42 to neuroprotective Aβ40, using experimental models containing wild-type (WT) and *PSEN1*-deficient fibroblasts. Altered glycosylation was observed in ACE purified from *PSEN1*-deficient fibroblasts while the Aβ42-to-Aβ40 conversion rate was reduced compared to WT counterparts. The reduced conversion ability of ACE was further found to be age-dependent, since adult brains demonstrated lower conversion capability. These results highlight the crucial contribution of PSEN1 in keeping low the levels of neurotoxic Aβ42 rendering it as an important target for AD therapeutic strategies.

One of the greatest genetic factors for AD and related dementias (ADRDs) is the *APOE*^ε4^allele (Zhao et al., 2020). Foley et al., aimed to delineate its contribution employing a new set of humanized *APOE* mouse models. The authors aimed to point out the unique effects of the heterozygote phenotype (*APOE*^ε3/ε4^*)* in the pathogenetic mechanisms observed in ADRDs. Differential contributions of the two main genotypes (*APOE*^ε3/ε4^ and *APOE*^ε4/ε4^) on cortical gene expression were observed. These results highlight the importance of the *APOE*ε*4* allele dosage-specific effects in the selection of the optimal therapy for clinical routine in patients suffering from AD-related dementia.

The phospholipid-transporting ATP binding cassette subfamily A member 7 (ABCA7) gene has been shown to have strong associations with AD (Stepler et al., 2022). In a case control study conducted by Wang L. et al. the association of a set of single nucleotide polymorphisms (SNPs) with AD was explored particularly for patients from southern China. The study detected 21 selected SNPs using molecular techniques. The ABCA7 rs3764650 SNP was positively correlated with AD morbidity. However, another ABCA7 SNP, rs4147929, was related to higher AD risk. These results suggest the involvement of *ABCA7* SNPs in AD and highlight the requirement for studies with larger patient cohorts.

Bartoletti-Stella et al. aimed to further identify pathogenic variants in genes as predictive risk factors for early-onset AD (EOAD). Using Next-Generation Sequencing (NGS) the authors assessed a panel of causal and risk factor genes for AD and dementia in Italian patients. Almost 11% of patients carried pathogenic or likely pathogenic variants in *PSEN1, PSEN2*, and *APP* while almost 8% of patients were homozygous for the ε4 *APOE* allele. Additionally, a significant fraction of patients carried only a variant in genes associated with other NDs. Therefore, genetic screening in EOAD patients could assist in their prognosis unraveling the pathogenic mechanisms that take place in AD and other forms of dementia.

In a last study for AD and related diseases, Liu et al. re-analyzed clinical data from literature from familial AD patients aiming to identify correlations between likely pathogenic amyloid precursor protein (APP) mutations, a major pathogenic gene for AD (Hinz and Geschwind, 2017), and phenotypical traits. Common clinical features such as amnesia, impairment in cognitive function and behavioral and psychological disorders were consistent with typical AD. The authors further reported different clinical manifestations depending on whether patients carried *APP* or *PSEN1/PSEN2* mutations thus potentially contributing to earlier clinical prognosis.

Loss-of-function mutations in *RAB39B* gene, a GTPase involved in intracellular vesicle trafficking (Cheng et al., 2002), are associated with early-onset Parkinson's disease (EOPD). Characteristic features of PD pathology, such as substantial loss of dopaminergic neurons in the substantia nigra and widespread Lewy body pathology were associated with Rab39b knockdown (Wilson et al., 2014). Wang Z. et al. aimed to further elucidate the role of RAB39B in PD pathogenesis. A typical PD model was induced by 1-methyl-4-phenyl-1,2,3,6-tetrahydropyridine (MPTP) to Rab39b knock-out (KO) mice. Both WT and Rab39b KO mice showed the same impairment in their motor activity and the same loss of dopaminergic neurons suggesting that RAB39B deficiency has no additive contribution to PD-like pathogenesis.

Aquaporin-4 (AQP4) plays a central role in the glymphatic system facilitating macromolecules draining to the interstitial fluid contributing to waste clearance in the brain (Mestre et al., 2018). Among the waste removed by AQP4, α-synuclein and Aβ are included, thus linking its function with PD (Zou et al., 2019) and AD (Iliff et al., 2012) pathologies. Fang et al. studied the association of AQP4 SNPs with motor and cognitive function in PD patients as well as the levels of neurotoxic species in the CSF. The AQP4 rs162009 SNP was associated with slower dementia conversion and better cognitive functions, lower Aβ deposition in different brain regions, while higher CSF levels of Aβ42 were observed in the subgroup of patients with REM sleep behavior disorder (RBD). The latter supports the notion that PD patients with sleep disorders demonstrate concomitant disruption of the glymphatic system (Bohnen and Hu, 2019). These results render the rs162009 SNP as an effective genetic prognostic marker for cognitive decline in PD patients potentially due to a malfunction in brain waste clearance system facilitated by sleep disruptions.

Rossi et al. aimed to identify the associated genetic variants of the semantic and right temporal variants of frontotemporal dementia (svFTD and rtvFTD). Twelve pathogenic or likely pathogenic variants were found in almost 40% of patients. These mutations were localized in genes implicated in processes such as autophagy. More importantly, these mutations may overlap with other neurodegenerative conditions such as AD and amyotrophic lateral sclerosis (ALS) indicating common pathogenetic mechanisms.

Thrombospondin-1 (THBS1) plasma levels have been shown to be elevated in ischemic stroke (IS) patients (Gao et al., 2015). Chen et al. evaluated the association of *THBS1* SNPs and the mRNA expression of THBS1 with the risk as well as the long-term outcome of IS. The authors reported no significant contribution of genotype and haplotype frequencies of rs2236741 and rs3743125. Risk of IS incidence or long-term death was not associated with any of the THBS1 variants. No further association was reported for *THBS1* mRNA expression levels as well, questioning the importance of THBS-1 in IS outcomes.

We believe that the present Research Topic articles highlights the important contribution of genetic inheritance in the pathogenetic malfunctions observed in NDs providing novel insights that need further research.

## Author contributions

IHP: Writing—review and editing. DA: Writing—review and editing. SR: Writing—original draft, Writing-review and editing.
